# Preliminary exploration of the ability of HUC-MSCs to restore the lung microbiota and related metabolite disorders in IPF treatment: combining 16S sequencing and metabolite analysis

**DOI:** 10.3389/fmicb.2025.1645577

**Published:** 2025-09-18

**Authors:** Shuang Zhou, Yukai Luo, Jun Liu, Jishui Huang, Xiaojing Zhang, Qing-chun Jia, Yijian Lin, Zhenyong Huang, Yiming Zeng, Wenzhao Cheng

**Affiliations:** ^1^Fujian Key Laboratory of Lung Stem Cells, The Second Affiliated Hospital of Fujian Medical University, Quanzhou, Fujian, China; ^2^The Second Clinical Medical School of Fujian Medical University, Quanzhou, Fujian, China; ^3^Department of Respiratory and Critical Care Medicine, The Second Hospital of Longyan, Longyan, Fujian, China; ^4^Department of Respiratory and Critical Care Medicine, The Hospital of Nanan City, Quanzhou, Fujian, China; ^5^Jinan Microecological Biomedicine Shandong Laboratory, Jinan, Shandong, China; ^6^Respiratory Medicine Center of Fujian, The Second Affiliated Hospital of Fujian Medical University, Quanzhou, Fujian, China

**Keywords:** IPF, HUC-MSCs, 16S rDNA sequence, lung microbiota, metabolites

## Abstract

**Background:**

Idiopathic pulmonary fibrosis (IPF) is a chronic and progressive pulmonary disease, and effective therapies to reverse the natural course of IPF are lacking. A growing number of studies have shown that the use of human umbilical cord-derived mesenchymal stem cells (HUC-MSCs) is a promising therapeutic strategy. However, the mechanism by which HUC-MSCs alleviate IPF and how HUC-MSCs affect the lung microbiota are still unclear and need further exploration.

**Methods:**

Bleomycin (BLM) injection was used to establish a mouse model of IPF, and 16S rDNA sequencing and LC–MS/MS metabolomics were performed to explore the underlying mechanism of HUC-MSCs as IPF treatment. Thirty mice were allocated into three groups, namely, the Control, BLM, and BLM + HUC-MSCs groups, and lung morphology; levels of α-SMA, FN1 and COL1A1; and levels of the inflammatory cytokines TNF-α, IL-1β, IL-6, and TGF-β1 were evaluated. Bronchoalveolar lavage fluid (BALF) samples from six mice in each of the three groups were collected randomly for 16S rDNA sequencing to analyze the lung microbiota and untargeted metabolomics analysis.

**Results:**

Human umbilical cord-derived mesenchymal stem cells restored alveolar morphology and reduced the expression of α-SMA, FN1 and COL1A1 and the inflammatory cytokines TNF-α, IL-1β, IL-6, and TGF-β1 in IPF model mice, confirming the anti-inflammatory properties of HUC-MSCs in IPF treatment. The 16S rDNA sequencing results indicated that HUC-MSCs treatment effectively decreased α diversity indices, such as the Abundance-based Coverage Estimator (ACE) and Shannon indices, as well as β diversity, leading to a decrease in microbiota abundance. The metabolomics analysis revealed that the metabolites exhibiting notable differences included primarily organic acids and their derivatives, lipids and lipid-like molecules, phenylpropanoids and polyketides, and organic nitrogen compounds, indicating the potential of HUC-MSCs to exert antifibrotic effects through these metabolic pathways.

**Conclusion:**

Overall, our study preliminarily confirmed that IPF in mice was closely related to microbial and metabolic dysbiosis. In mice with IPF, treatment with HUC-MSCs modulated dysregulated metabolic pathways and improved microbiota function to a state more comparable to that of the Control group. This study provides new insights into the potential mechanisms and treatments of IPF.

## 1 Introduction

Pulmonary fibrosis (PF) is a chronic progressive disease characterized by the deposition of large amounts of extracellular matrix (ECM), fibroblast proliferation, inflammatory infiltrative damage and destruction of alveolar structures (Heukels et al., 2019). Idiopathic pulmonary fibrosis (IPF) is a type of PF with low survival and low cure rates, with overall incidence and prevalence estimates of 0.09–1.30 and 0.33–4.51 per 10,000 people, respectively (Lu et al., 2024). Its progression is often related to patient age and sex, genetic and environmental factors, the progression of lung inflammation, and the lung microbiota (Savin et al., 2022). Despite the consistent development of diagnostic and therapeutic options for IPF in recent years and subsequent improvements in patient survival, therapies other than lung transplantation that can cure IPF are still lacking (Bonella et al., 2023). Therefore, the search for potential treatments for IPF has become an urgent issue.

With advances in medical treatment, stem cells are gradually becoming a “panacea” for these incurable diseases. Mesenchymal stem cells (MSCs) are known for their proliferative capacity, multilinear differentiation, and immunomodulatory capacity (Ding et al., 2015), attracting substantial interest. The extraction process for human umbilical cord-derived MSCs (HUC-MSCs) has the advantages of being painless and simple to perform compared with that for bone marrow MSCs (BM-MSCs). In recent years, an increasing number of studies have demonstrated the potential of HUC-MSCs in the treatment of respiratory diseases, such as asthma, chronic obstructive pulmonary disease (COPD), and acute respiratory distress syndrome (ARDS) (Bukreieva et al., 2023; Lanzoni et al., 2021; Sharan et al., 2023). Although previous studies have indicated a role for HUC-MSCs in the treatment of IPF (Mahmoudi et al., 2020; Zhang et al., 2023), changes in lung microbes and their metabolites have not been reported.

The microbiota has a reciprocal symbiosis with the human body and is widely present in the oral cavity, gastrointestinal tract, skin, and respiratory tract (Yang et al., 2020). With advances in sampling techniques and characterization, the traditional belief that the lungs are sterile is no longer true. The microbiota of the lungs is much smaller than that of the gastrointestinal tract and consists of four phyla: *Firmicutes, Proteobacteria, Bacteroidetes*, and *Actinomycetes*; this distribution is also observed in mice (Dickson et al., 2016). 16S rDNA sequencing is a powerful tool for characterizing the lung microbiota. Recent study has shown that the diversity and composition of the microbiota in patients with IPF are associated with increased levels of alveolar profibrotic cytokines (O’Dwyer et al., 2019). Another study showed that the pulmonary microbiota *Bacteroides* and *Prevotella* contribute to fibrosis pathogenesis through IL-17R signaling and exacerbate bleomycin (BLM)-induced PF (Yang et al., 2019). In addition, SiO_2_ and PM_2.5_ inhalation are strongly associated with lung microbiota disorders (Utembe and Kamng’ona, 2024; Zhou et al., 2024). These results suggest that changes in the lung microbiota play a crucial role in PF progression, suggesting that dysregulation of lung ecology may be a necessary trigger. Although the progression of IPF and the associated changes in the lung microbiota have been investigated in previous studies, the effects of changes in the lung microbiota in IPF patients treated with HUC-MSCs have not been reported.

The development and maturation of multiomics technologies have enabled a more in-depth understanding of disease progression (Karczewski and Snyder, 2018). Metabolomics provides the most comprehensive information about the functional state of cells and aids in the characterization of the phenotypes of organisms, providing new insights for exploring the underlying mechanisms of disease and identifying effective therapeutic targets (González-Domínguez et al., 2015). Recent studies have shown that several metabolic pathways involved in the metabolism of lipids, proteins, and carbohydrates can disrupt intercellular functions (such as proliferation, signaling, and death) and the body’s metabolic homeostasis and promote IPF progression (Liang et al., 2024; Rajesh et al., 2023). Although many previous studies have elucidated metabolite alterations in IPF, those in IPF patients treated with HUC-MSCs have not been reported, and the role of metabolite alterations in the treatment of PF with HUC-MSCs has not been fully established.

In this study, changes in the lung microbiota in BLM-induced lung fibrosis was investigated via 16S ribosomal DNA (16S rDNA) amplification sequencing to determine the role of the lung microbiota in the modulation of BLM-induced lung inflammation and fibrosis through HUC-MSCs intervention. Metabolite changes were subsequently investigated via untargeted metabolomics sequencing. The aim of this study was to comprehensively reveal the relationship between dysregulation of the lung microbiota and changes in metabolic profiles by integrating microbiomic and metabolomic analyses and to provide new insights for the early prevention and anti-inflammation of PF.

## 2 Materials and methods

### 2.1 Isolation and identification of HUC-MSCs

The neonatal umbilical cords used for the isolation of HUC-MSCs were obtained from healthy donors according to standard protocols (Xiang et al., 2020). Briefly, the umbilical cord was cleaned and cut into 1–2 cm^3^ pieces. These tissue blocks were cultured in Dulbecco’s modified Eagle’s medium (DMEM)/F12 medium (Thermo Fisher Scientific, No. 21331020) containing 10% fetal bovine serum (FBS, Thermo Fisher Scientific, No. A5670701) and stored at 37 °C in a 5% CO_2_ humidified incubator. The primary culture medium was changed after 3 days to remove non-adherent cells. Afterward, the medium was renewed every 2 days. Fibroblast-like cell colonies appeared after 14 days, after which the cells were trypsinized and transferred to new flasks for further expansion. P3 HUC-MSCs were used for phenotypic and pluripotent property assessment and experimental analysis. In terms of safety, comprehensive testing has been conducted. This includes screening for aerobic and facultative anaerobic bacteria, obligate anaerobic bacteria, and fungi, as well as endotoxin testing and Gram staining verification to ensure that there is no bacterial contamination. Each donor signed an informed consent form, and the Second Affiliated Hospital ethics committee of Fujian Medical University approved the protocol for umbilical cord harvesting (No. 20210395).

### 2.2 Animals

Thirty 6-weeks-old male C57BL/6 mice were purchased from SLAC Laboratory Animal Co., Ltd., (Shanghai, China). The mice were housed and maintained in a controlled environment with a temperature of 24°C ± 2°C, humidity of 50% ± 5%, and a regular light cycle of 12 h of light and 12 h of dark; adequate food and water were provided. Animal experiments were conducted in accordance with the guidelines of the Center for Animal Experimentation and approved by the Ethics Committee of the Second Affiliated Hospital of Fujian Medical University (Approval No. 20210395).

### 2.3 Animal experimental treatment and HUC-MSCs administration

Thirty mice were allowed to acclimatize to the environment for 1 week and then randomly divided into three groups (*n* = 10 per group). The first group was the Control group, in which the mice were not treated with any factor; the second group was the BLM group, in which the mice were anesthetized with sodium pentobarbital, BLM was infused intratracheally through a 21G non-invasive intubation at a dose of 1 U/kg, and the mice were turned evenly so that BLM was perfused uniformly in the lungs. The third group was the BLM + HUC-MSCs group; 1 week after the mice received the BLM infusion, 10 mice were randomly selected, the posture and position of the mice were immobilized with a fixator, and 5 × 10^5^ cells (2.5 × 10^7^ cells/kg) were injected through the tail vein of the mice with a syringe. At the endpoint (day 14), the mice were euthanized with an overdose of 3% sodium pentobarbital, and every effort was made to minimize discomfort and pain during the process.

### 2.4 Sample collection and histological analysis

After the mice were anesthetized with 3% sodium pentobarbital, we collected blood through the apices of the mice for the determination of cytokine levels in the serum. Before serum collection, the collected blood was left to sit at room temperature for 3 h, followed by centrifugation at 3000 rcf for 20 min at 4°C to collect the serum, which was placed in sterile tubes and stored at −80°C (*n* = 10). To obtain BALF samples, we dissected the mice and ligated the left lung (*n* = 6); collected the alveolar lavage fluid from the right lung by adding ice-cold sterile PBS (1,000 μL) into the right airway dropwise; rinsed the airway three times; and centrifuged the collected BALF for 30 min at 4°C at 10,000 rpm. The collected supernatant was used to determine inflammatory cytokine levels, and the precipitate was snap-frozen in liquid nitrogen and immediately stored at −80°C for 16S rDNA sequencing analysis. The ligated left lung was removed and stored at −80°C for untargeted metabolomics analysis. Bilateral lungs from the remaining mice (*n* = 4) were also harvested. First, the mice were irrigated three times with 500 μL of PBS and injected with 500 μL of 4% paraformaldehyde for fixation. After fixation for 24 h and dehydration with an alcohol gradient, the lung tissues were embedded in paraffin. The tissue blocks were cut into 5 μm sections from the frontal, middle and posterior coronal planes and stained with hematoxylin and eosin (HE). HE-stained sections were used for Ashcroft scoring (Hübner et al., 2008). All the mice were anesthetized and sacrificed with 3% sodium pentobarbital after the test, and every effort was made to minimize discomfort and pain.

### 2.5 Immunostaining

Tissue sections were obtained as described previously. The sections were subsequently dewaxed, dehydrated and repaired with sodium citrate under high temperature and pressure. The samples were incubated with primary antibodies for 1 h at room temperature or overnight at 4°C. The primary antibodies used were as follows: anti-α-SMA (Abcam, ab124964), anti-FN1 (Abcam, ab2413), and anti-COL1A1 (Abcam, ab260043). The samples were then incubated with the secondary antibody for 2 h at room temperature. The samples were then washed three times with PBST, and the nuclei were stained with DAPI for 10 min. Finally, an anti-fluorescence quencher was added to the sections for neutral dendrimer sealing and imaging under a DMI8 confocal microscope (Leica, Germany).

### 2.6 Cytokine level measurement with ELISA

The levels of IL-1β, IL-6, TNF-α, and TGF-β1 in the serum and BALF were determined with ELISA (R&D Systems, USA, IL-1β: VAL601, IL-6: VAL604G, TNF-α: VAL609, TGF-β1: VAL611).

### 2.7 Measurement of hydroxyproline content

The hydroxyproline assay is widely regarded as a gold-standard for assessing fibrotic burden in experimental models. To determine the hydroxyproline content, the mice were dissected to obtain the lung and the blood were drained. According to the manufacturer’s instructions (A030-2-1, Jiancheng Bioengineering Institute, Nanjing, China), we weighed 50 mg of wet mouse lung tissue, added the hydrolysis solution, and placed it at 95°C for thorough hydrolysis, adjusting it to the preset PH value (6.0–6.8). After thorough mixing, the solution was incubated at 60°C for 15 min, cooled, and then centrifuged at 3,500 rpm for 10 min at room temperature. The absorbance of each sample at 550 nm was measured, and the hydroxyproline content was calculated using the formul.

### 2.8 16S rDNA gene sequencing analysis

Microbial DNA was isolated using a HiPure Stool DNA Kit according to the manufacturer’s instructions. The 16S rDNA V3–V4 region was amplified via PCR (95°C for 5 min; 30 cycles of 95°C for 1 min, 60°C for 1 min and 72°C for 1 min; and a final extension at 72°C for 7 min) using the universal forward and reverse primers 341F (CCTACGGGNGGCWGCAG) and 806R (GGACTACHVGGGTATCTAAT). Amplicons were collected from 2% agarose gels, purified with an AxyPrep DNA Gel Extraction Kit (Axygen Biosciences, Union City, CA, USA) according to the manufacturer’s instructions, and quantified using the ABI Step One Plus Real-Time PCR System (Life Technologies, Foster City, USA). The purified amplicons were sequenced on the standard Illumina platform for double-end sequencing (PE 250) according to the standard procedure. The raw reads were further filtered with FASTP (version 0.18.0). Paired-end clean reads were merged as raw tags with FLASH (version 1.2.11), with a minimum overlap of 10 bp and mismatch error rates of 2%. The raw tags were then quality filtered, and the chimeric sequences were removed to acquire the effective tags, which were clustered into operational taxonomic units (OTUs) according to a ≥97% similarity cutoff with UPARSE software (version 9.2.64). Principal coordinates analysis (PCoA) was performed with the R Project Vegan software package (version 2.5.3). The observed species, Abundance-based Coverage Estimator (ACE) index and Shannon diversity index were obtained with QIIME software (version 1.9.1, University of Colorado, Denver, CO, USA) and used to analyze the effects of BLM and HUC-MSCs on the overall microbiota structure. Alpha index comparisons between multiple groups were performed via the Kruskal–Wallis H test using the R language Vegan package (version 2.5.3). Dunn’s test was used for post hoc pairwise comparisons in the Kruskal-Wallis H test. Venn analysis was performed with the R language VennDiagram package (version 1.6.16) and UpSetR package (version 1.3.3) to analyze the shared intergroup OTUs as well as endemic OTUs. The biomarker species in each subgroup were screened with LEfSe software (version 1.0), the random forest package (version 4.6.12), the pROC package (version 1.10.0), and the Labdsv package (version 2.0.1). Ternary maps of species abundance were generated with the R package ggtern (version 3.1.0). Kyoto Encyclopedia of Genes and Genomes (KEGG) metabolic pathway analysis of sample bacteria or archaea was performed with Tax4Fun (version 1.0). The functional differences between groups were analyzed via Welch’s *t*-test using the R language Vegan package (version 2.5.3). The heatmap of cluster stacking was calculated with the R package and generated by OmicsMart (Gene Denovo Biotechnology Co., Ltd., Guangzhou, China), a dynamic real-time interactive platform for data analysis.

### 2.9 Metabolomic analysis: LC-MS/MS untargeted metabolomic analysis

Samples were thawed slowly at 4°C, and an appropriate amount of sample was added to precooled methanol/acetonitrile/water solution (2:2:1, v/v), vortexed, mixed, sonicated at low temperature for 30 min, and centrifuged at 14000 *g* at 4°C for 20 min. The supernatant was removed and dried under vacuum, and 100 μL of acetonitrile and water (acetonitrile: water = 1:1, v/v) was added to redissolve the sample for mass spectrometry. For mass spectrometry analysis, 100 μL of acetonitrile solution (acetonitrile: water = 1:1, v/v) was added to the sample, which was redissolved, vortexed, and centrifuged at 14000 × *g* for 15 min. Quality control (QC) samples were mixed with equal volumes from the samples to be tested and used to determine instrument status, balance the chromatography–mass spectrometry system before injection, and evaluate system stability during the experiment (Supplementary Figure 3). Subsequently, LC-MS/MS analysis was performed on the test samples.

The samples were separated on an Agilent 1290 Infinity LC ultrahigh-performance liquid chromatography (UHPLC) HILIC column; the column temperature was 25°C; the flow rate was 0.5 mL/min; the injection volume was 2 μL; and the mobile phases consisted of A: water + 25 mM ammonium acetate + 25 mM ammonia and B: acetonitrile. The gradient elution program was as follows: 0–0.5 min, 95% B; 0.5–7 min, B decreased linearly from 95% to 65%; 7–8 min, B decreased linearly from 65% to 40%; 8–9 min, B was maintained at 40%; 9–9.1 min, B increased linearly from 40% to 95%; and 9.1–12 min, B was maintained at 95%. The samples were placed in an autosampler at 4°C throughout the analysis. To avoid the influence of fluctuations in the instrumental detection signal, the samples were analyzed continuously in a random order. QC samples were inserted into the sample queue for monitoring and evaluating the stability of the system and the reliability of the experimental data.

The ESI source conditions after HILIC chromatographic separation were as follows: Ion Source Gas1: 60, Ion Source Gas2: 60, curtain gas (CUR): 30, source temperature: 600°C, ion spray floating voltage: ±5500 V (positive and negative modes), time-of-flight (TOF) MS scan m/z range: 60–1000 Da, product ion scan m/z range: 25–1000 Da, TOF MS scan accumulation time: 0.20 s/spectrum, product ion scan accumulation time: 0.05 s/spectrum, and secondary mass spectra: obtained via information-dependent acquisition (IDA) in high-sensitivity mode. The raw data were converted to the MzML format by ProteoWizard, and the XCMS program was used for peak alignment, retention time correction, and peak area extraction. The XCMS software parameters were set as follows: peak picking: centWave m/z = 10 ppm, peak width = c (10, 60), and prefilter = c (10, 100); peak grouping: b = 5, mzwid = 0.025, and minfrac = 0.5. The data obtained from XCMS extraction were checked for completeness, metabolites with more than 50% missing values within the group were removed from subsequent analysis, nulls were k-nearest neighbor (K-NN)-populated, extreme values were deleted, and total peak area was normalized to ensure that parallelism between samples and metabolites could be compared.

### 2.10 Statistical analysis

The data are presented as the means ± standard deviations (SDs). The significance of the differences was determined via one-way analysis of variance (ANOVA) or the Brown-Forsythe test. For normally distributed data, ANOVA is typically used for statistical difference analysis; for data that do not conform to a normal distribution, the Brown-Forsyth test is used. Histograms and dot plots were created with Graph Pad Prism 9 software (San Diego, CA, USA). Bioinformatics analysis, including species taxonomy, richness and diversity analyses, was performed with OmicsMart (Guangzhou, China). The correlations between the lung microbiota and metabolites were analyzed via Spearman correlation tests. A *p*-value < 0.05 indicated a statistically significant difference.

## 3 Results

### 3.1 Identification of HUC-MSCs phenotypes

**FIGURE 1:**
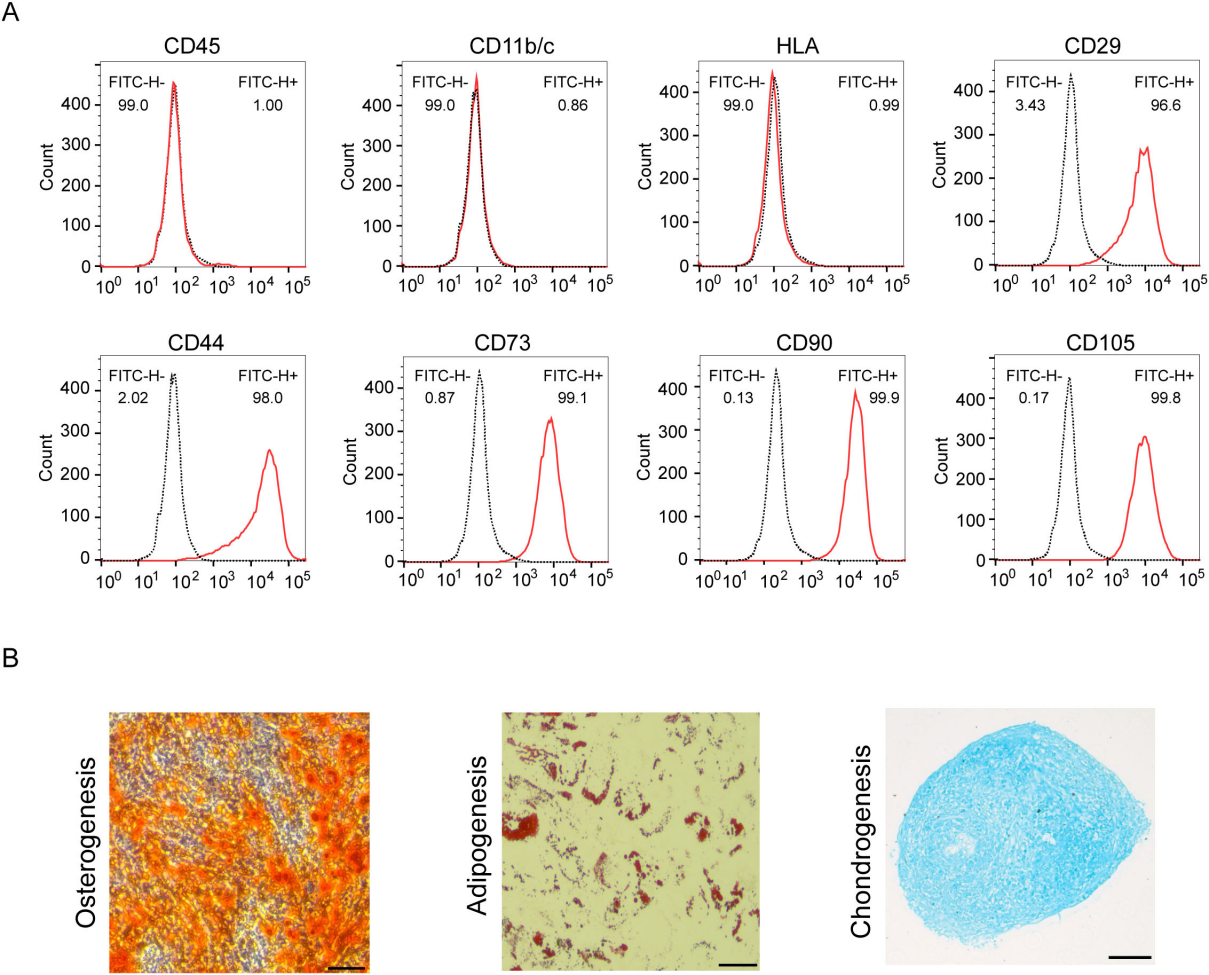
Identification of HUC-MSCs. **(A)** Cells were analyzed by flow cytometry with the following antibodies: anti-CD11b/c, anti-CD45, anti-HLA, anti-CD29, anti-CD44, anti-CD73, anti-CD90, and anti-CD105. **(B)** Determination of the potential of HUC-MSCs for adipogenic, chondrogenic and osteogenic differentiation (scale bar: 100 μm).

Mesenchymal stem cells were isolated from human umbilical cords and cultured until a fibroblast-like morphology was obtained. HUC-MSCs expressed specific antigens, including CD29^+^, CD44^+^, CD73^+^, CD90^+^, and CD105^+^, and did not express the hematopoietic spectrum markers CD11b/c, CD45, and HLA (Figure 1A). In addition, we evaluated the trilineage differentiation ability of HUC-MSCs and HUC-MSCs were successfully induced to differentiate into osteoblasts, chondrocytes and adipocytes (Figure 1B).

### 3.2 Attenuation of the severity of BLM-induced IPF after HUC-MSCs administration in model mice

**FIGURE 2:**
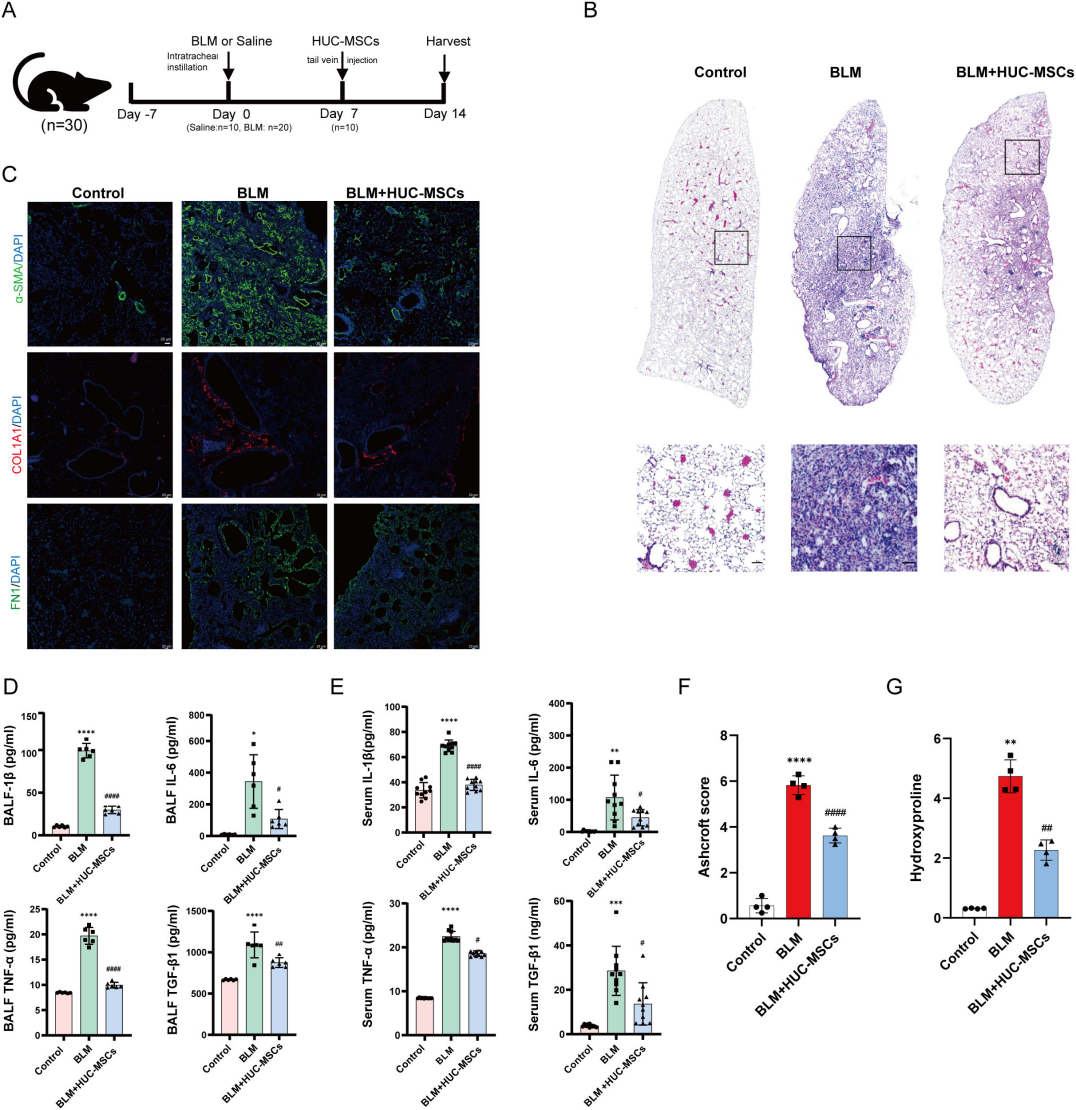
Human umbilical cord-derived mesenchymal stem cells (HUC-MSCs) alleviated the severity of BLM-induced IPF in model mice. **(A)** Experimental design and model schematic. **(B)** Representative HE-stained lung sections from mice (scale bar: 50 μm). **(C)** Representative images of immunofluorescence staining and the expression of α-SMA, FN1 and COL1A1 in lung tissues (scale bar: 20 μm). **(D,E)** The concentrations of IL-1β, IL-6, TNF-α and TGF-β1 in bronchoalveolar lavage fluid (BALF) and serum were evaluated with ELISA kits. **(F)** Ashcroft score. **(G)** The concentrations of Hydroxyproline. The data are shown as the means ± SDs (Figure 1D, *n* = 6; Figure 1E, *n* = 10; Figure 1F, *n* = 4) (*p < 0.05, **p < 0.01, ***p < 0.001, ****p < 0.0001 BLM vs. Control; ^#^*p* < 0.05, ^##^*p* < 0.01, ^###^*p* < 0.001, ^####^*p* < 0.0001 vs. BLM).

In the current study, the mechanism underlying changes in the lung microbiota during BLM-induced PF and HUC-MSCs administration post-BLM exposure, which occurred on day 14, were investigated (Figure 2A). Histopathological staining was used to evaluate the histological characteristics of the lung samples. Compared with those in the Control group, the alveolar structure, alveolar wall thickening, alveolar lumen compression and inflammatory cell infiltration were disrupted in the BLM group. The application of HUC-MSCs improved lung conditions, as evidenced by better protection of alveolar integrity, less pronounced alveolar wall thickening, and reduced inflammatory cell infiltration (Figure 2B). In addition, we calculated the Ashcroft score to analyze the HE staining results. The results indicated that the Ashcroft score was significantly lower in lung tissues from the BLM + HUC-MSCs group than in those from the BLM group (Figure 2F). α-SMA, COL1A1 and FN1 are biomarkers for assessing fibrosis (Livingston et al., 2023). Immunofluorescence was used to assess the expression of α-SMA, COL1A1 and FN1 in lung tissue. The results revealed that these three indicators were almost undetectable in the Control group, whereas in the BLM group, the expression of COL1A1 and FN1 was significantly elevated in the peritracheal area, and the expression of α-SMA was concentrated in the interstitium of the lungs and the peritracheal area; however, after treatment with HUC-MSCs, the expression levels of these biomarkers were downregulated (Figure 2C). In addition, the hydroxyproline content in the lungs of mice increased significantly after BLM administration, while after treatment with HUC-MSCs, the hydroxyproline content decreased significantly (Figure 2G). In summary, these results highlight the protective effects of HUC-MSCs on the development of BLM-induced PF.

### 3.3 Effects of HUC-MSCs administration on inflammatory cytokine levels in the serum and BALF

To analyze the healing effect of HUC-MSCs on lung inflammation, the expression levels of inflammatory cytokines, including IL-1β, IL-6, TNF-α and TGF-β1, in the serum and BALF were measured using ELISA. The cytokine levels in the serum were significantly greater in the BLM groups than in the Control group, and the cytokine levels in the BALF exhibited a similar trend. In addition, compared with those in the BLM group, cytokine levels in the BALF were significantly lower in the BLM + HUC-MSCs group after the administration of HUC-MSCs (Figures 2D, E).

### 3.4 Alteration of the lung microbiota structure and distribution in BLM-induced IPF model mice after HUC-MSCs administration

**FIGURE 3:**
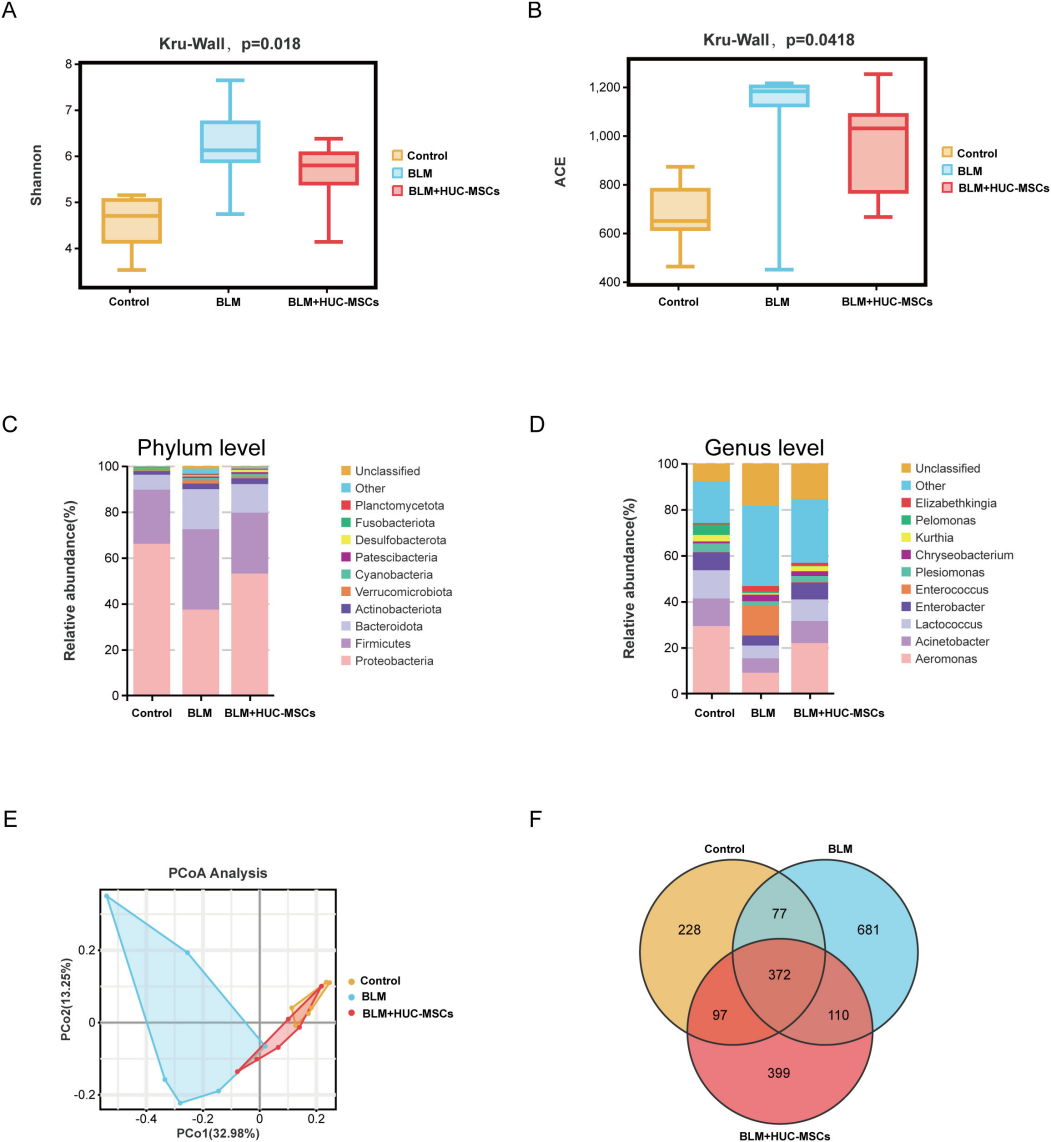
Human umbilical cord-derived mesenchymal stem cells (HUC-MSCs) therapy altered the lung microbiota composition in BLM-induced IPF model mice. **(A)** Shannon richness index of the lung microbiota. **(B)** ACE richness index of the lung microbiota. **(C)** Relative abundances of bacterial taxa in all the groups at the phylum level. **(D)** Relative abundances of bacterial taxa in all the groups at the genus level. **(E)** PCoA plot of microbial communities at the OTU level. **(F)** Venn diagram showing the distribution of operational taxonomic units (OTUs) among different groups. The data are shown as the means ± SDs (*n* = 6 for all the groups).

To explore the effect of HUC-MSCs on the structure of the lung microbiota of BLM-induced IPF mice, the lung microbiota was assessed via 16S rDNA sequencing. As shown in Figures 3A, B, the ACE and Shannon indices tended to increase after BLM treatment but decreased in response to the administration of HUC-MSCs. In addition, we compared the composition of the lung microbiota among the three groups of mice at the phylum and genus levels to better assess the efficacy of HUC-MSCs administration in BLM-induced IPF model mice. At the phylum level, *Proteobacteria*, *Firmicutes*, and *Bacteroidetes* were the major components of the lung microbiota. Compared with those in the Control group, the relative abundances of *Proteobacteria* and *Fusobacteriota* significantly decreased with the administration of BLM, whereas the abundances of *Firmicutes*, *Bacteroidota* and *Verrucomicrobiota* increased. The administration of HUC-MSCs increased the proportion of *Proteobacteria* and decreased the proportions of *Firmicutes*, *Bacteroidota*, and *Verrucomicrobiota* (Figure 3C). At the genus level, *Aeromonas*, *Acinetobacter*, and *Lactococcus* were the main components of the lung microbiota. Compared with those in the Control group, *Aeromonas*, *Enterobacter*, *Lactococcus* and *Kurthia* were significantly lower in the model group, whereas *Elizabethkingia* was relatively lower with BLM administration. However, the administration of HUC-MSCs increased the proportions of *Aeromonas*, *Enterobacter*, *Lactococcus* and *Kurthia*. The proportion of *Elizabethkingia* decreased in the HUC-MSCs treatment group (Figure 3D). The β diversity plot indicated aggregation within each of the three groups, with distinct positions and shapes observed between the groups. As shown in Figure 3E, the PCoA plot indicated a close similarity of the lung microbiota in the Control and BLM + HUC-MSCs groups. In addition, the composition of the lung microbiota in the BLM group was distinct from that in the other two groups. Venn diagram analysis revealed that the three groups shared 372 OTUs, with 228, 681 and 399 unique OTUs in the Control, BLM and BLM + HUC-MSCs groups, respectively (Figure 3F). Details on the box plots and heatmaps of the 10 most abundant microbiota at the phylum and genus levels is provided in the Supplementary Material (Supplementary Figures 1, 2). In summary, these results demonstrated that HUC-MSCs could alleviate lung microbiota disorders in BLM-induced IPF model mice.

### 3.5 Analysis of microbiota differences among the three groups

**FIGURE 4:**
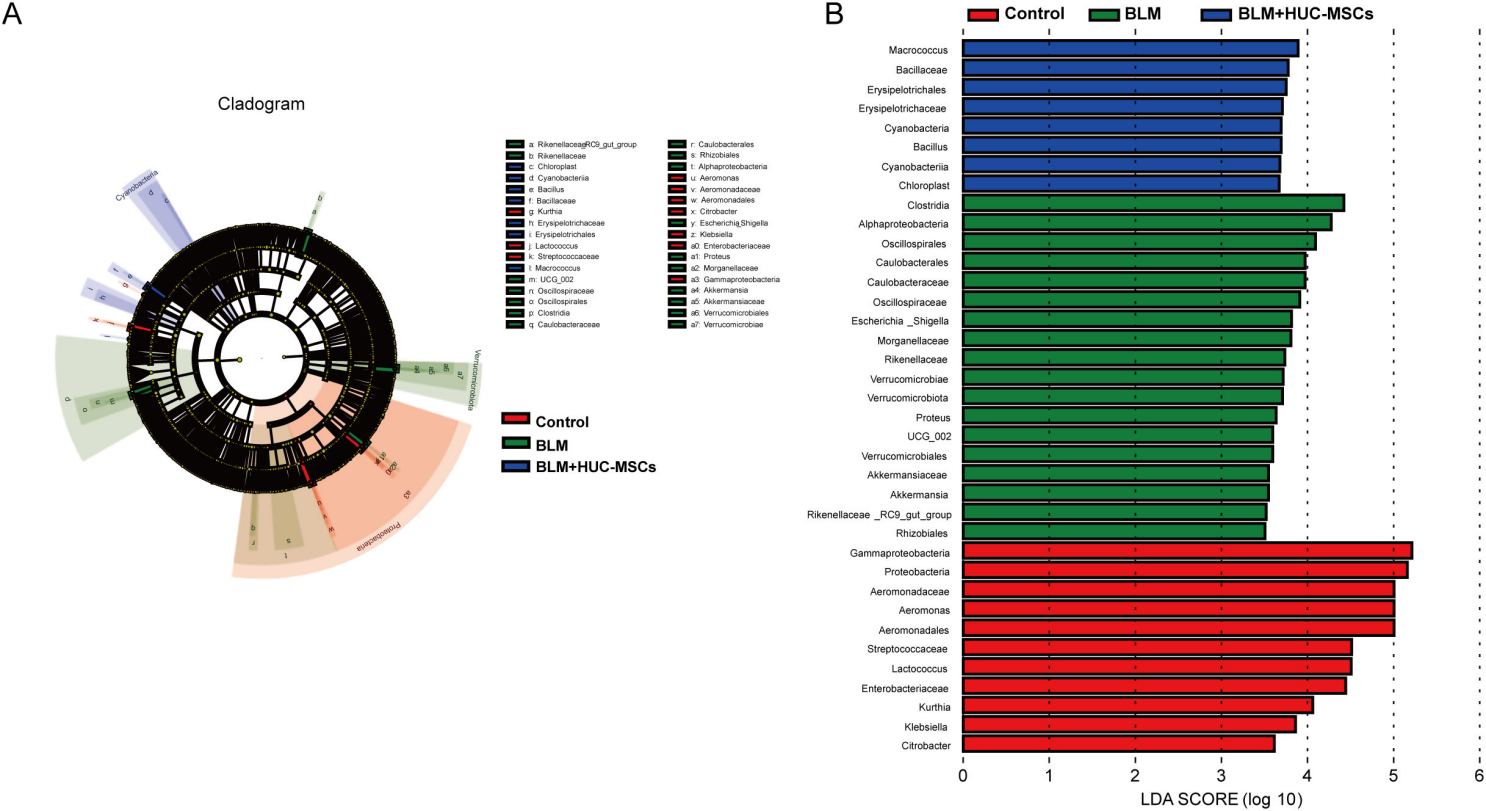
LEfSe analysis. **(A)** Cladogram. The circles radiating from the inside out represent the classification level from boundary to genus. **(B)** Histogram of the LDA value distribution. The longer the length is, the greater the degree of influence.

To gain better insight into the impact of HUC-MSCs intervention on the lung microbiota of mice, we conducted linear discriminant analysis size effect (LefSe). As shown in Figures 4A, B, biomarkers with linear discriminant analysis (LDA) values > 3 and *p* < 0.05 were screened. γ-*Protobacteria*, *Proteobacteria*, *Aeromonadaceae*, *Aeromonas*, *Aeromonadales* and *Streptococcaceae* were enriched in the Control group. *Clostridia*, α-*Proteobacteria*, *Oscillospirales*, *Caulobacteraceae*, *Oscillospiraceae* and *Caulobacterales* were enriched in the BLM group. The BLM + HUC-MSCs group presented increased relative abundances of *Macrococcus*, *Bacillaceae, Erysipelotrichales, Cyanobacteria, Bacilli*, and *Erysipelotrichaceae*.

### 3.6 Prediction of lung microbiota function and the correlation with cytokine levels

**FIGURE 5:**
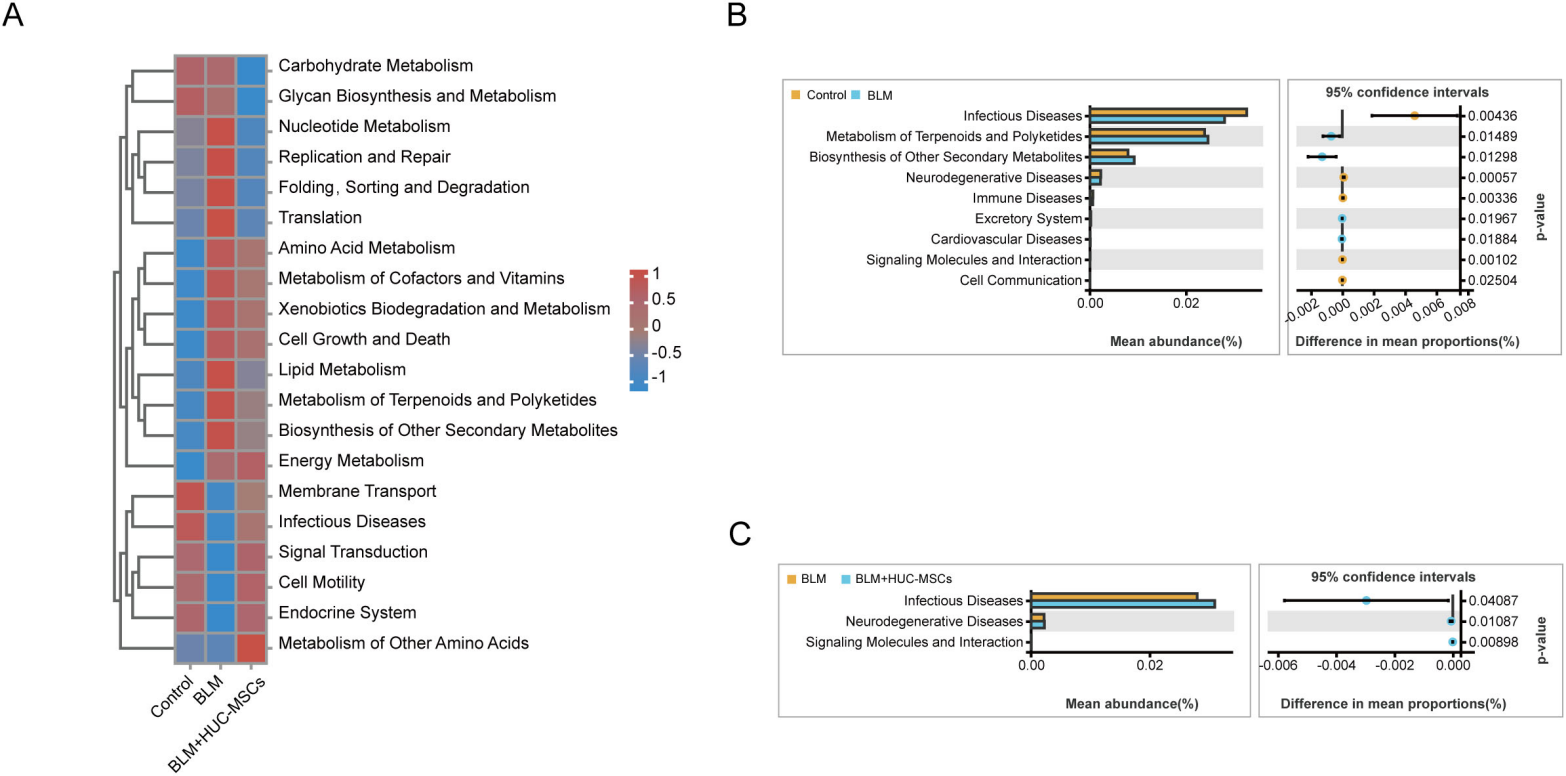
Analysis to predict the metabolic function of the lung microbiota on the basis of Tax4Fun predictions. **(A)** Heatmap of the level 2 functional pathways of the lung microbiota. **(B)** Comparison of microbial function prediction between the Control group and the BLM group. **(C)** Comparison of microbial function prediction between the BLM group and the BLM+HUC-MSCs group.

To explore the changes in lung microbiota function in HUC-MSCs-treated IPF model mice, KEGG functions were annotated on the basis of the Tax4Fun analysis. Clustered heatmaps from the KEGG analysis revealed significantly different metabolic pathways within the lung microbiota when the mice were subjected to different factors (Figure 5A). BLM substantially downregulated the activity of pathways associated with membrane transport, infectious disease, signal transduction, cell motility and the endocrine system. In turn, the activity of these metabolic pathways was significantly upregulated by the administration of HUC-MSCs. Pathways with significantly upregulated activity after BLM exposure and downregulated activity after treatment with HUC-MSCs were involved in nucleotide metabolism, replication and repair, folding, sorting and degradation, translation and lipid metabolism. Conversely, the pathways with significantly upregulated activity after BLM exposure but no downregulated activity after HUC-MSCs treatment were amino acid metabolism, metabolism of cofactors and vitamins, cell growth and death, biosynthesis of other secondary bile acids, energy metabolism and xenobiotic biodegradation and metabolism.

A two-sided Welch’s *t*-test between pairs of means was used to compare the predicted functional pathways in the lung microbiota among the three groups. In the BLM group, the activity of pathways associated with infectious diseases, neurodegenerative diseases and immune diseases were decreased. However, BLM increased the metabolism of terpenes and polyketides and the biosynthesis of other secondary metabolites. HUC-MSCs administration upregulated the activity of pathways associated with infectious diseases and neurodegenerative diseases (Figures 5B, C). In summary, these results suggested that HUC-MSCs likely altered lung microbiota function in BLM-induced IPF model mice.

Spearman correlation analysis was used to investigate the relationships between the lung microbiota and inflammatory factors. At the genus level (Figure 8A), IL-1β was negatively correlated with *Aeromonas* (*r* = −0.72, *p* < 0.001), *Lactococcus* (*r* = −0.65, *p* < 0.01), and *Kurthia* (*r* = −0.54, *p* < 0.05) but positively correlated with *Elizabethkingia* (*r* = 0.49, *p* < 0.05). TNF-α was negatively correlated with *Aeromonas* (*r* = −0.74, *p* < 0.001), *Lactococcus* (*r* = −0.70, *p* < 0.01), and *Kurthia* (*r* = −0.65, *p* < 0.01) but positively correlated with *Enterococcus* (*r* = 0.48, *p* < 0.05) and *Elizabethkingia* (*r* = 0.48, *p* < 0.05). TGF-β1 was negatively correlated with *Aeromonas* (*r* = −0.82, *p* < 0.001), *Lactococcus* (*r* = −0.82, *p* < 0.001), *Kurthia* (*r* = −0.71, *p* < 0.01), and *Enterobacter* (*r* = −0.50, *p* < 0.05) but positively correlated with *Elizabethkingia* (*r* = −0.68, *p* < 0.05). IL-6 was negatively correlated with *Aeromonas* (*r* = −0.68, *p* < 0.01), *Lactococcus* (*r* = −0.73, *p* < 0.001), and *Kurthia* (*r* = −0.72, *p* < 0.001). These results suggest that lung microbiota may influence the expression and secretion of cytokines involved in inflammation and immunity.

### 3.7 Identification of key metabolites in the HUC-MSCs treatment group

**FIGURE 6:**
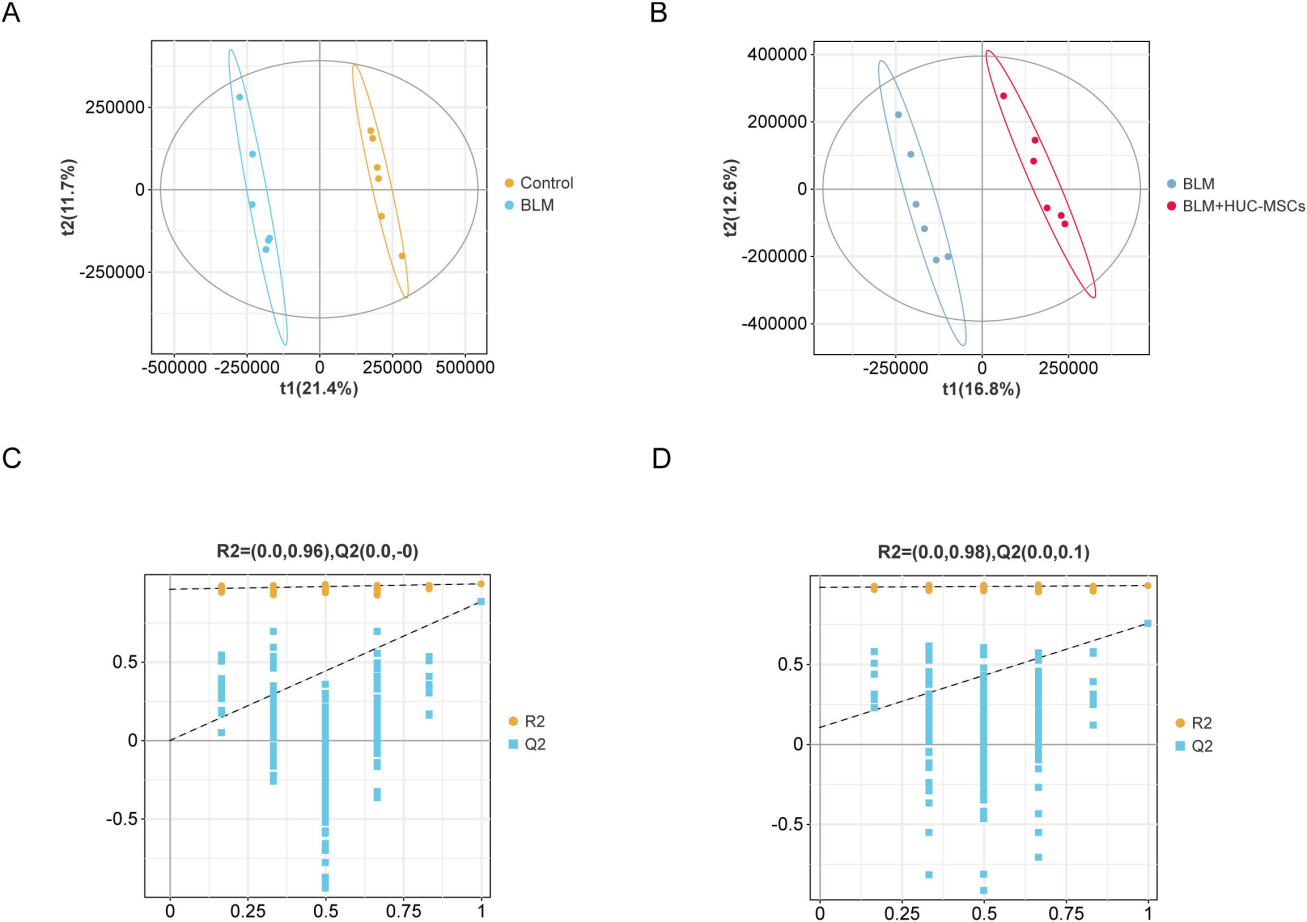
Score plots for the PLS-DA model and its corresponding permutation test plots. **(A)** PLS–DA diagrams of the Control and BLM groups. **(B)** PLS–DA diagrams of the BLM and BLM + HUC-MSCs groups. **(C–D)** Corresponding model validation plots.

The results of partial least squares discriminant analysis (PLS-DA) indicated that the samples of each group could be clearly separated from each other, suggesting a dissimilar metabolic mode (Figures 6A, B). The model was subsequently verified via a permutation test. R2 and Q2 were lower than the rightmost original values from left to right, indicating that the model had good predictive ability (Figures 6C, D).

**FIGURE 7:**
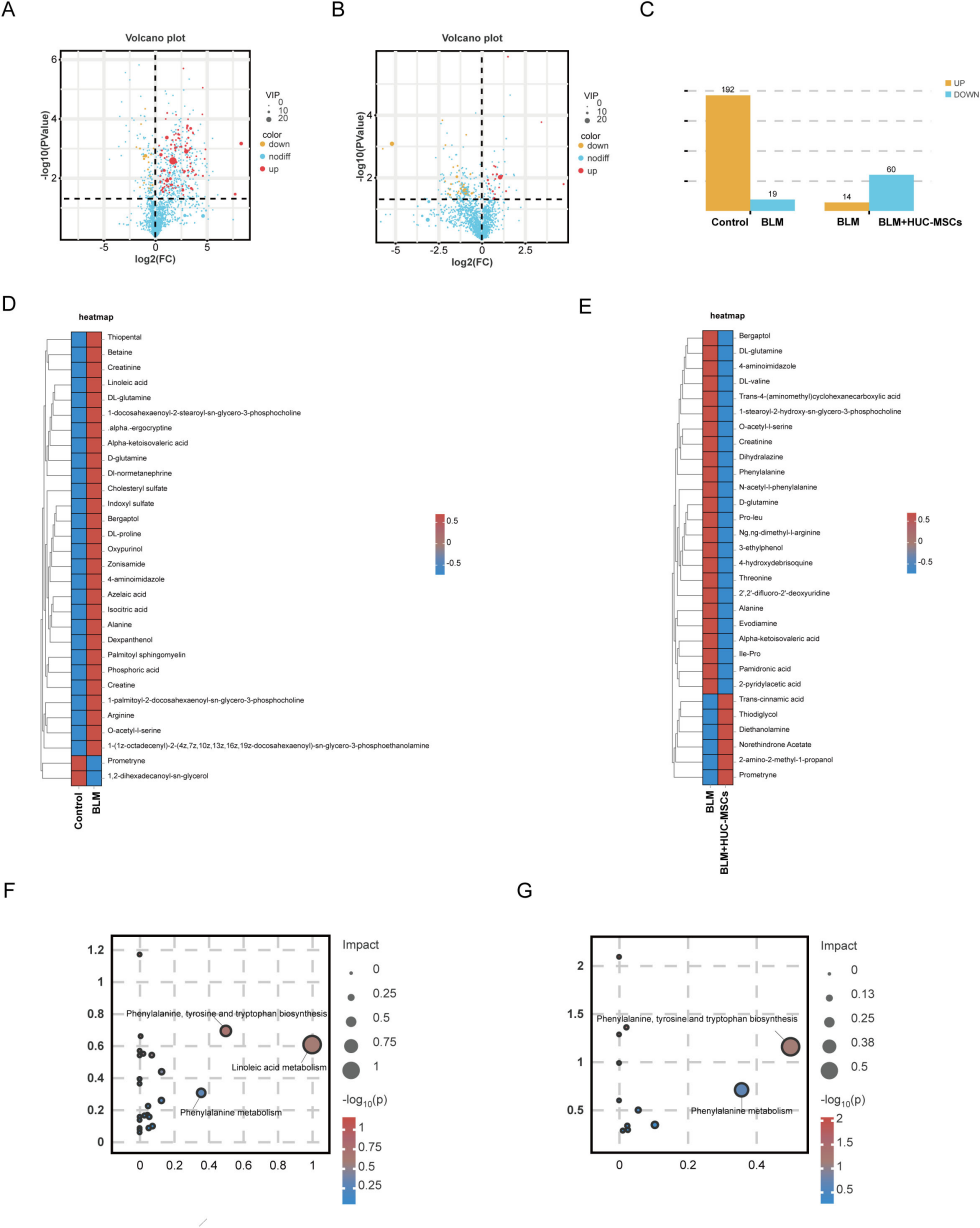
Differentially abundant metabolite identification and enrichment analysis based on the KEGG database. **(A)** Volcano plots of differentially abundant metabolites between the Control and BLM groups. **(B)** Volcanic map of differentially abundant metabolites between the BLM and BLM + HUC-MSCs groups. **(C)** Histogram of changes in differentially abundant metabolites. **(D,E)** Heatmap of the clustering of differentially abundant metabolites. The colors from red to blue in the heatmap indicate the abundance of metabolites in each group of samples from high to low. **(F)** KEGG pathway analysis of differentially abundant metabolites between the Control and BLM groups. **(G)** KEGG pathway analysis of differentially abundant metabolites between the BLM and BLM + HUC-MSCs groups.

Metabolites were analyzed by PLS-DA and volcano plots to identify the differentially abundant metabolites (Figures 7A, B). The metabolites that differed significantly between the BLM group and the Control group and between the BLM group and the BLM + HUC-MSCs group, according to the criteria of variable importance in projection (VIP) > 1, *P* < 0.05, and FC > 2 or FC < 0.5 (log_2_ FC > 1 or log_2_ FC < −1), were selected and compared; subsequently, the Human Metabolome Database (HMDB) was used to validate the differentially abundant metabolites. The numbers of significantly different metabolites in the Control group versus the BLM group and the BLM group versus the BLM + HUC-MSCs group are shown in Figure 7C. A total of 211 eligible differentially abundant metabolites were identified in the BLM group compared with the Control group, of which 192 metabolites were upregulated and 19 metabolites were downregulated, and a total of 74 eligible differentially abundant metabolites were identified in the BLM group compared with the BLM + HUC-MSCs group, of which 14 metabolites were upregulated and 60 metabolites were downregulated. We selected the top 30 differentially abundant metabolites ranked by VIP value for heatmap analysis. This analysis revealed that in the BLM group, 28 differentially abundant metabolites were significantly upregulated and 2 metabolites were downregulated compared with those in the Control group, and 24 differentially abundant metabolites were significantly downregulated and 6 metabolites were upregulated compared with those in the BLM + HUC-MSCs group (Figures 7D, E). The metabolites included organic acids and their derivatives, lipids and lipid-like molecules, phenylpropanoids and polyketides, and organic nitrogen compounds.

To investigate the changes caused by metabolite differences, we analyzed the metabolites that were significantly different between the Control group and the BLM group using the KEGG database and selected the metabolic pathways that had an IMPACT value greater than 0.2. Three significantly different metabolic pathways, including linoleic acid metabolism; phenylalanine, tyrosine and tryptophan biosynthesis; and phenylalanine metabolism, were identified. Of these, phenylalanine, tyrosine and tryptophan biosynthesis and phenylalanine metabolism are the main pathways affected by HUC-MSCs treatment (Figures 7F, G).

### 3.8 Correlation analysis

**FIGURE 8:**
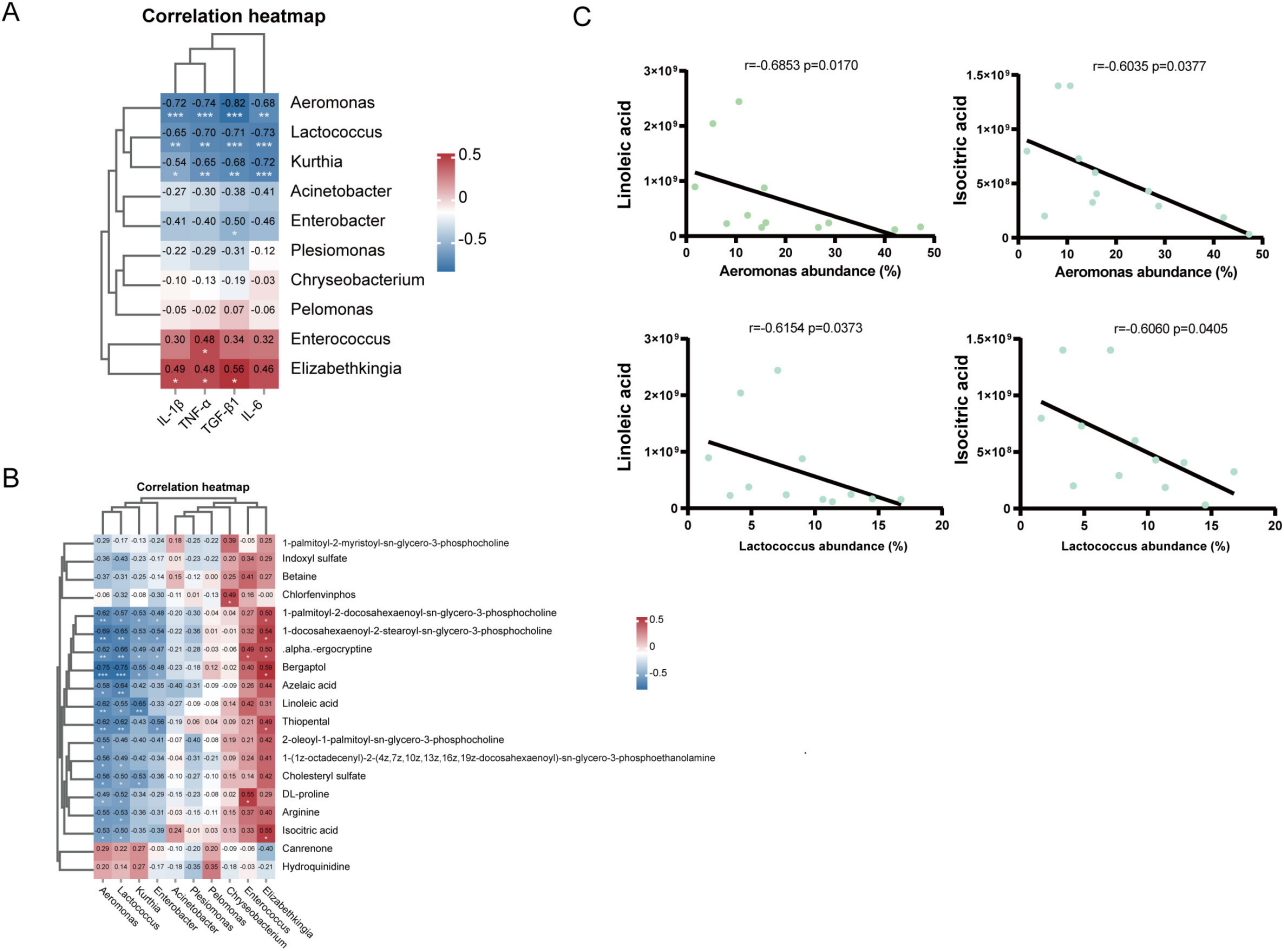
Correlations among the lung microbiota and cytokines and metabolites. **(A)** Spearman analysis of cytokines and 10 key differentially abundant genes in the lung microbiota at the genus level. **(B)** Spearman analysis of 20 significantly different metabolites and 10 key differentially abundant metabolites in the lung microbiota at the genus level. **(C)** Spearman’s rank correlation between the relative abundances of *Aeromonas*, *Lactococcus* and several metabolites (*p < 0.05, **p < 0.01, ***p < 0.001, ****p < 0.0001).

To further explore the relationships between changes in microbiota abundance and alterations in metabolite composition, we used Spearman correlation analysis to investigate the relationships between the microbiota at the genus level and differentially abundant metabolites. Correlation heatmaps revealed an association between the lung microbiota and metabolites (Figure 8B). These results indicated that the abundance of *Aeromonas* and *Kurthia* decreased significantly after BLM exposure, whereas the contents of linoleic acid and isocitric acid increased. Therefore, the correlations between *Aeromonas, Kurthia* and linoleic acid and isocitric acid were investigated (Figure 8C). *Aeromonas* was negatively correlated with linoleic acid (*r* = −0.6853, *p* = 0.0170) and isocitric acid (*r* = −0.6620, *p* = 0.0223) and was negatively correlated with linoleic acid (*r* = −0.7622, *p* = 0.0055). The results of this study suggest that changes in the lung microbiota are associated with alterations in metabolites and that there may be an interaction between these two factors that promotes the progression of PF.

## 4 Discussion

In this study, we established a mouse model of BLM-induced IPF. BLM administration elicits an inflammatory and fibrotic response in a short period, causing lung tissue changes similar to those in IPF patients; BLM administration is also easy to perform and reproduce, so this method is widely used in animal models of IPF (Moeller et al., 2008). The advent of 16S rDNA sequencing has opened new avenues for elucidating the pathophysiologic role of the microbiota in a wide range of host diseases (Thapa et al., 2024). Interestingly, compared with the gut microbiota, the lung microbiota is less abundant and dynamically present (Ramírez-Labrada et al., 2020). Lung microbiota dysbiosis is correlated with alveolar inflammation, indicators of PF, and disease progression in animal models (O’Dwyer et al., 2019). Notably, a clinical study revealed that *Streptococcus* and *Staphylococcus* in the lungs were associated with IPF progression (Han et al., 2014). The application of MSCs can mitigate the progression of diseases such as colitis and pulmonary hypertension by modulating the immune response and microbiota composition (Luo et al., 2021; Wang et al., 2023). However, changes in the lung microbiota during the treatment of IPF with MSCs have not been reported. Therefore, in the present study, an animal model of IPF was constructed via intra-airway titration of BLM, and an intervention with HUC-MSCs was performed to alter the lung microbiota in mice with PF and assess the role of the lung microbiota in this disease. The immunofluorescence results for the fibrosis markers α-SMA, FN1, and COL1A1; the HE staining results for lung morphology; the hydroxyproline content and the ELISA results for the inflammatory factors IL-1β, IL-6, and TNF-α and the profibrotic factor TGF-β1 demonstrated that treatment with HUC-MSCs attenuated lung fibrosis compared with that in the BLM group. However, due to the insufficient modeling time in mice, these results are more indicative of the role of HUC-MSCs in preventing the progression of pulmonary fibrosis and their anti-inflammatory effects. Further studies with additional and longer time points are needed to demonstrate their ability to reverse pulmonary fibrosis. In words, these results suggest that dysregulation of lung microorganisms plays an important role in BLM-induced PF and provide new insights into BLM-induced PF and developing strategies to combat it.

The 16S rDNA sequencing results revealed that the Shannon and ACE indices increased in BLM-exposed mice and decreased after treatment with HUC-MSCs, suggesting that HUC-MSCs can improve α diversity in mice, indicating an increase in the diversity and abundance of microorganisms in the lungs. The β diversity results revealed that the microbes in the BLM group differed significantly from those in the Control and BLM + HUC-MSCs groups, whereas the microbes in the Control group partially overlapped with those in the BLM + HUC-MSCs group, suggesting that HUC-MSCs can, to some extent, correct the disruption of the lung microbiota induced by PF. Our study revealed that the major phyla in healthy lungs were *Proteobacteria, Firmicutes* and *Bacteroides*, which is consistent with the findings of previous studies (Dickson et al., 2014; Segal et al., 2013). After BLM treatment, the relative abundance of *Proteobacteria* decreased significantly, and the relative abundances of Bacteroides and *Verrucomicrobiota* increased significantly. After treatment with HUC-MSCs, the relative abundance of *Proteobacteria* increased significantly. At the genus level, *Aeromonas*, *Lactococcus*, and *Acinetobacter* were the dominant organisms; the relative abundances of *Aeromonas*, *Lactococcus*, *Enterobacter*, and *Kurthia* significantly decreased in the BLM group, and the relative abundances of *Lactococcus* and *Enterobacter* significantly increased after treatment with HUC-MSCs in the BLM + HUC-MSCs group. *Proteobacteria* is considered the largest phylum in the bacterial structural domain because of the presence of lipopolysaccharides in its outer membrane, leading to negative gram-staining results (Rizzatti et al., 2017). Several previous studies have shown that the progression of respiratory disease is associated with an increased abundance of *Proteobacteria*. However, a recent study based on sequencing of 16S rDNA from the BALF of IPF patients revealed a decreasing trend in the relative abundance of *Proteobacteria*, and this finding was validated in a BLM-challenged animal model (Takahashi et al., 2018). The results of our study are consistent with those of previous studies. However, the specific role *Proteobacteria* plays in IPF has not been elucidated. *Lactococcus* belongs to the *Firmicutes* phylum and is widely found in the human gastrointestinal tract, where it functions as a “probiotic” in the human body (Goldstein et al., 2015). *Lactococcus* produces metabolites with anti-inflammatory properties, such as short-chain fatty acids, to modulate immune responses, including the activation of dendritic cells and Th1 lymphocytes and a reduction in Th2 lymphocyte responses (Amrouche et al., 2024). In addition, previous studies have shown that the application of *Lactobacillus* can attenuate the progression of ALI (Shen et al., 2023), reduce intestinal permeability and decrease proinflammatory cytokines in individuals with colitis through biofilm formation (Qiu et al., 2023). In our study, the decrease in *Lactococcus* may have been due to an inflammatory response in the body that led to an increase in intestinal permeability, allowing *Lactococcus* to reach the gastrointestinal tract for mucosal repair through the lung–gut axis. However, further investigations are needed to fully elucidate the underlying mechanism involved. *Enterobacter* is a genus of common gram-negative, partially anaerobic, rod-shaped, non-spore-forming bacteria belonging to the family *Enterobacteriaceae* (Davin-Regli and Pagès, 2015). In previous studies, an increased abundance of *Enterobacter* was associated with severe gastrointestinal disease and bacterial resistance; however, in a recent study exploring COPD in mice, the abundance of *Enterobacter* was greater in the non-smoking group than in the smoking group (Zhang et al., 2018). Furthermore, an increased abundance of *Enterobacter* appears to be positively associated with survival in severe pneumonia patients (Huang et al., 2024). Our study revealed a decreased abundance of *Enterobacter* in the lungs of BLM-treated mice. However, the specific mechanisms influencing changes in the abundance and distribution of *Enterobacteria* under different physiological conditions remain unclear and require further experimentation to elucidate the potential mechanisms that may influence these changes. Nevertheless, there are differences between the lung microbiota of mice and humans, and there are different therapeutic response effects between different species (O’Dwyer et al., 2019). A recent study showed that HUC-MSCs significantly increased IFN-γ in alveolar lavage fluid in mouse models, while this effect was weaker in human clinical trials (Li et al., 2025). Whereas, cross-species validation of the regulation of lung microbiota changes by HUC-MSCs still requires more refined models and more realistic disease environments and immune microenvironments to confirm. In conclusion, alterations of the lung microbiota disrupt the reciprocal symbiotic relationship between the microbiota and the host, and HUC-MSCs may effectively inhibit inflammatory cell infiltration and lung fibrosis by regulating the lung microbiota.

In our study, we investigated changes in lung metabolites after BLM injury and after intervention with HUC-MSCs via LC–MS untargeted metabolomics analysis. The results indicated that the metabolite changes were focused mainly on organic acids and derivatives, lipids and lipid-like molecules, and phenylpropanoids and polyketides. The abundances of linoleic acid, isocitric acid, D-glutamine, DL-glutamine, phenylalanine, and N-phenylalanine were significantly increased after BLM injury compared with those in the Control group, and the abundances of phenylalanine, N-phenylalanine, DL-glutamine and D-glutamine were significantly decreased after intervention with HUC-MSCs. KEGG enrichment analysis revealed that the differentially abundant metabolites after BLM exposure were involved mainly in linoleic acid metabolism; phenylalanine, tyrosine and tryptophan biosynthesis; and phenylalanine metabolism, whereas the differentially abundant metabolites after HUC-MSCs administration were involved mainly in phenylalanine, tyrosine and tryptophan biosynthesis and phenylalanine metabolism. The Tax4Fun predictions revealed an increase in the abundance of lipid and amino acid metabolites in the BLM group and the BLM + HUC-MSCs group, suggesting that metabolite alterations are consistent with microbial function predictions. Linoleic acid is a structural component of membrane phospholipids that maintains membrane fluidity. Linoleic acid is a precursor of arachidonic acid, which produces prostaglandins and leukotrienes, potentially mediating inflammation (Whelan and Fritsche, 2013). 13-S-Hydroxyoctadecadienoic acid (13-S-HODE), a metabolite of linoleic acid, induces mitochondrial dysfunction and airway epithelial cell apoptosis, which leads to severe airway obstruction, lung remodeling, and increased levels of epithelial stress-related proinflammatory cytokines in mice (Mabalirajan et al., 2013). A clinical trial of patients with COPD and IPF revealed that the patients had significantly greater levels of linoleic acid than did normal individuals (Mabalirajan et al., 2013). Increased linoleic acid content leads to the proliferation of TGF-β1-induced fibroblast lines and significantly enhances TGF-β1-induced α-SMA expression, further exacerbating PF (Kim et al., 2017), which is consistent with our experimental results; however, how linoleic acid and its metabolites affect IPF progression has not been elucidated. Isocitrate is an intermediate metabolite involved in glutaminolysis reactions, citrate export, conversion to α-ketoglutarate, and NADPH shuttling during metabolic stress and cell proliferation (Kim et al., 2017). Reduced levels of isocitrate, a circulating intermediate of the tricarboxylic acid (TCA) cycle, have been observed in the inflamed mucosa of patients with inflammatory bowel disease, and a positive effect of isocitrate in the treatment of early acute inflammatory anemia has been reported (Kim et al., 2016; Richardson et al., 2013). However, recent research pointed out that isocitric acid remained elevated during COPD exacerbations, which may be due to disruption of the TCA cycle (Kim et al., 2022), which seems to be consistent with our results; inconsistent changes in isocitric acid levels may be attributed to different organ environments and disease progression. Substantial previous evidence has suggested a critical role for amino acid metabolism in the progression of pulmonary fibrogenesis (Roque and Romero, 2021). Glutamine is the most abundant amino acid in blood (Cruzat et al., 2018) and is converted to glutamate in the presence of GSL1, thus participating in myofibroblast activation and differentiation (Choudhury et al., 2023). In IPF patients, the activity of enzymes involved in glutamine metabolism–GLS1, GOT2, OGDH and SUCLG–are reduced, leading to decreased levels of glutamine metabolism in mitochondria, which may result in glutamine accumulation. Inhibition of glutamine degradation impairs the differentiation and proliferation of AT2 cells, leading to impaired self-repair capacity and exacerbating pulmonary fibrosis (Nachef et al., 2021; Wang et al., 2022). In addition, glutamine is considered a nutritional immunomodulator that plays a key role in the immune system. In a previous study, G.G. Dos Santos discovered the role of glutamine metabolism in regulating MSC immune function. Increased glutamine metabolism promoted the production of the anti-inflammatory factor IL-10 and reduced the secretion of the pro-inflammatory factors IL-1β and IL-6 (Dos Santos et al., 2017; Li et al., 2023). This correlation could explain the increase in glutamine levels in lung tissue after BLM exposure and the decrease in glutamine levels after HUC-MSCs intervention. However, the specific mechanism has not yet been clarified. Phenylalanine is an essential amino acid that increases inflammation and immune responses and has been proposed as an indicator to predict disease severity in critical patients. Previous studies have shown that phenylalanine accumulation is associated with the progression of COVID-19 and ARDS (Luporini et al., 2021; Tang et al., 2023). The previous view was that the lungs do not use phenylalanine directly; however, a recent study by Tan et al. (2020) demonstrated that phenylalanine accumulates in the BALF after intravenous injection. These results provide basic evidence that phenylalanine directly interacts with lung cells. Taken together, these results suggest that the application of HUC-MSCs altered the abundance and enrichment pathways of metabolites in BLM-injured mice, and disturbances in metabolic activity were associated with the progression of lung fibrosis in these mice.

This research mainly explored the role of HUC-MSCs in preventing IPF progression and their anti-inflammatory effects, but did not investigate the specific mechanisms by which HUC-MSCs regulate the lung microbiota. This may be related to their inherent antibacterial and immune-modulating effects (Gao et al., 2025). The specific mechanism by which mesenchymal stem cells promote the colonization of beneficial bacteria may be associated with creating a favorable intestinal microenvironment. The antibacterial peptides produced by mesenchymal stem cells can selectively target pathogens, promote the survival and proliferation of intestinal epithelial cells, upregulate tight junction molecules, and maintain intestinal integrity (Pan et al., 2025). HUC-MSCs-derived extracellular vesicles (EVs) regulate the composition of the gut microbiota, particularly by increasing the abundance of norank_f__*Muribaculaceae*, and influence liver metabolic characteristics, thereby significantly improving liver injury (Yang et al., 2025). Exploring this mechanism will help enhance the depth and translational value of the research.

The present study has several limitations. First, due to technical restrictions, this research did not verify the specific role of the microbiota using microbiota transplantation in germ-free mice. These potential mechanisms warrant further exploration in future studies. Procedural and sequencing contamination remain concerns in studies based on low-biomass sequencing. Our protocol was optimized to minimize contamination, including sequencing multiple procedural and reagent controls, using a low-biomass protocol for DNA extraction, and randomizing sample processing. However, contamination cannot be completely ruled out, and the issue requires further resolution. In additional, the study lacked an evaluation of the efficacy of the Control + HUC-MSCs group. This may lead to some of the changes in the lung microbiota, metabolites, and immune environment directly driven by HUC-MSCs being overlooked. The inclusion of Control + HUC-MSCs group will help to more clearly attribute the effects to disease reversal or baseline adjustment. Insufficient sample size and lack of clinical samples are also among the limitations of this study. Future studies should expand the sample size and collect more comprehensive clinical samples to explore the underlying mechanisms.

## 5 Conclusion

By combining 16S rDNA sequencing and untargeted metabolomics, this study demonstrated that HUC-MSCs significantly ameliorated α- and β- diversity of the lung microbiota and reversed abnormal microbial abundance in BLM-injured mice, while also modulating dysregulated metabolic pathways in IPF model mice. These findings provide the evidence of HUC-MSCs-mediated structural remodeling of the lung microbiota in a pulmonary fibrosis model, offering new insights into the mechanisms of MSC-based therapy. However, the precise underlying mechanisms and clinical applicability require further validation.

## Data Availability

The original contributions presented in this study are publicly available. This data can be found here: https://www.ebi.ac.uk/metabolights/, accession number MTBLS12922; https://www.ncbi.nlm.nih.gov/, accession number PRJNA1310954.

## Supplementary material

Supplementary Figure 1 Human umbilical cord-derived mesenchymal stem cells (HUC-MSCs) alter the composition of the lung microbiota at the phylum level in BLM-induced IPF model mice. The relative abundances of **(A)** Proteobacteria, **(B)** Firmicutes, **(C)** Bacteroidota, **(D)** Cyanobacteria, **(E)** Actinobacteriota, **(F)** Verrucomicrobiota, **(G)** Desulfobacterota, **(H)** Patescibacteria, **(I)** Fusobacteriota, and **(J)** Planctomycetota. **(K)** Heatmap of cluster stacking at the phylum level. The data are expressed as the means ± SDs (*n* = 6 for all the groups) (**p* < 0.05, ***p* < 0.01, ****p* < 0.001, *****p* < 0.0001 BLM vs. Control; ^#^*p* < 0.05, ^##^*p* < 0.01, ^###^*p* < 0.001, ^####^*p* < 0.0001 vs. BLM).

Supplementary Figure 2 Human umbilical cord-derived mesenchymal stem cells (HUC-MSCs) alter the composition of the lung microbiota at the genus level in BLM-induced IPF model mice. The relative abundances of **(A)** Aeromonas, **(B)** Lactococcus, **(C)** Enterobacter, **(D)** Acinetobacter, **(E)** Plesiomonas, **(F)** Chryseobacterium, **(G)** Elizabethkingia, **(H)** Enterococcus, **(I)** Kurthia, and **(J)** Pelomonas. **(K)** Heatmap of cluster stacking at the genus level. The data are expressed as the means ± SDs (*n* = 6 for all the groups) (**p* < 0.05, ***p* < 0.01, ****p* < 0.001, *****p* < 0.0001 BLM vs. Control; ^#^*p* < 0.05, ^##^*p* < 0.01, ^###^*p* < 0.001, ^####^*p* < 0.0001 vs. BLM).

Supplementary Figure 3 PCA score plot of three different groups in positive and negative ion modes (including QC samples).
