# Supplementary figures and images for: Preliminary exploration of the ability of HUC-MSCs to restore the lung microbiota and related metabolite disorders in IPF treatment: combining 16S sequencing and metabolite analysis
Source: Front Microbiol. 2025 Sep 18;16:1645577. doi: 10.3389/fmicb.2025.1645577 (PMC12489257; doi:10.3389/fmicb.2025.1645577)

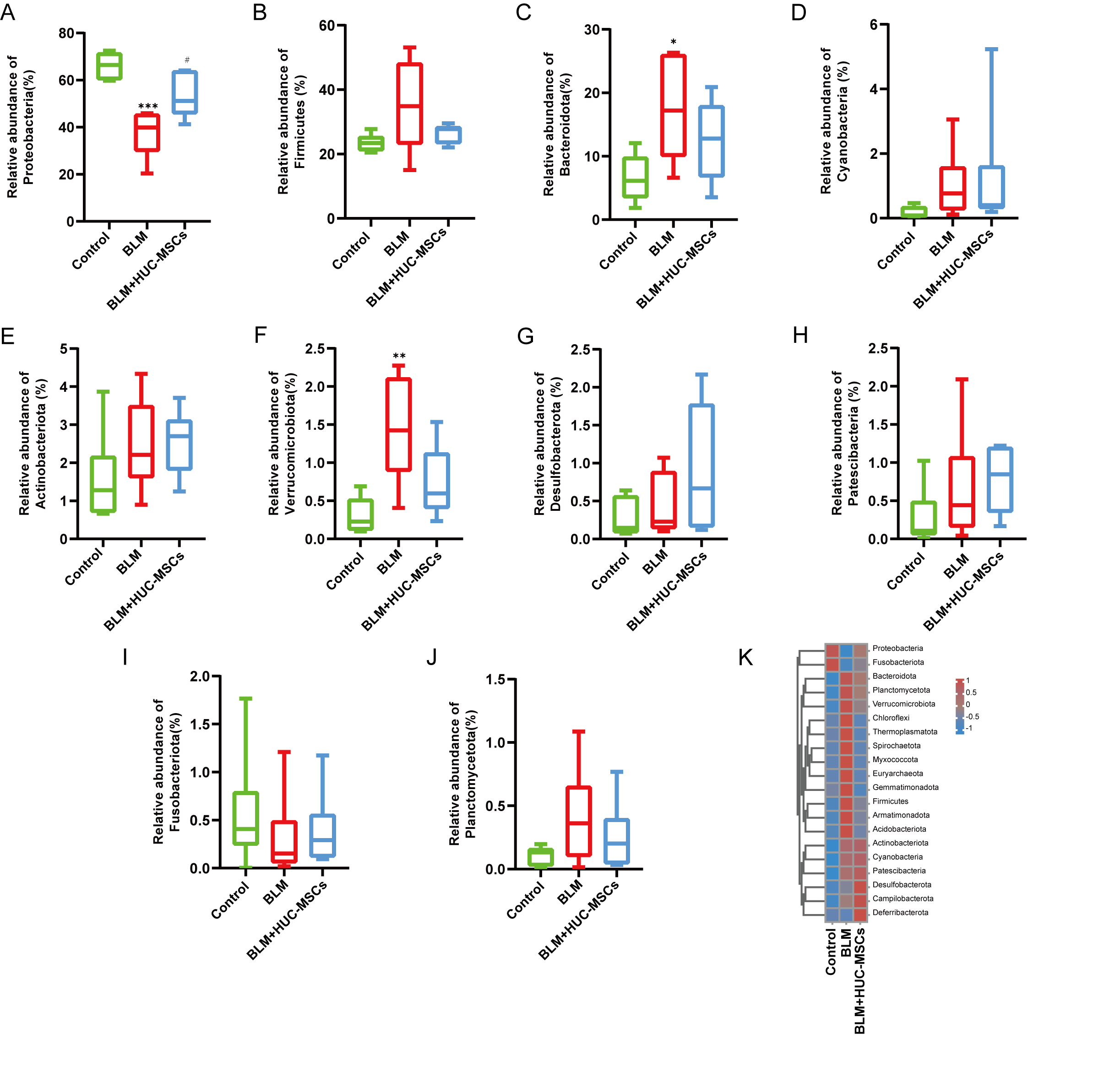

Supplement: Supplementary Figure 1 — Human umbilical cord-derived mesenchymal stem cells (HUC-MSCs) alter the composition of the lung microbiota at the phylum level in BLM-induced IPF model mice. The relative abundances of (A) Proteobacteria, (B) Firmicutes, (C) Bacteroidota, (D) Cyanobacteria, (E) Actinobacteriota, (F) Verrucomicrobiota, (G) Desulfobacterota, (H) Patescibacteria, (I) Fusobacteriota, and (J) Planctomycetota. (K) Heatmap of cluster stacking at the phylum level. The data are expressed as the means ± SDs (n = 6 for all the groups) (*p < 0.05, **p < 0.01, ***p < 0.001, ****p < 0.0001 BLM vs. Control; #p < 0.05, ##p < 0.01, ###p < 0.001, ####p < 0.0001 vs. BLM). [file Image_1.tif]

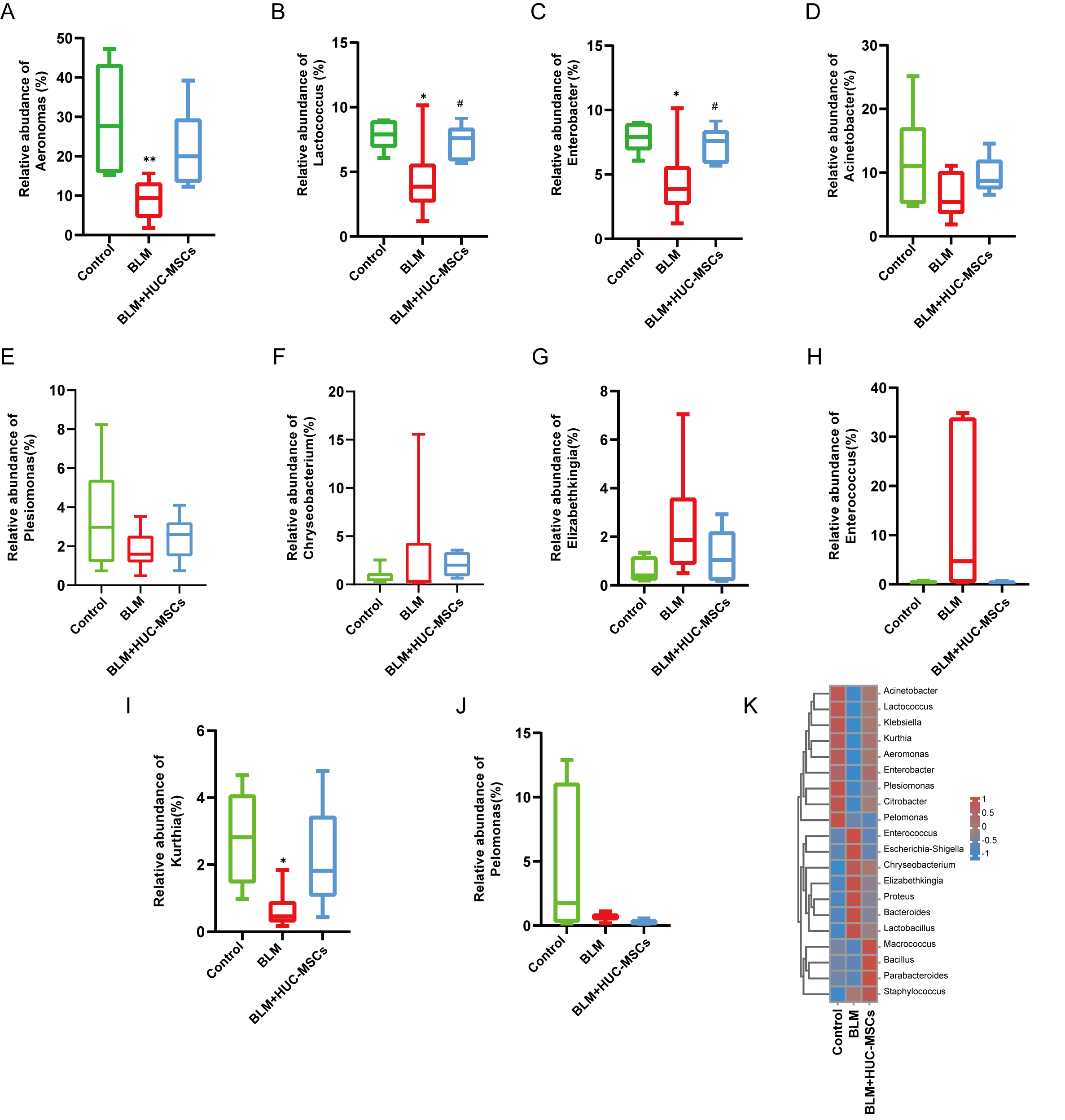

Supplement: Supplementary Figure 2 — Human umbilical cord-derived mesenchymal stem cells (HUC-MSCs) alter the composition of the lung microbiota at the genus level in BLM-induced IPF model mice. The relative abundances of (A) Aeromonas, (B) Lactococcus, (C) Enterobacter, (D) Acinetobacter, (E) Plesiomonas, (F) Chryseobacterium, (G) Elizabethkingia, (H) Enterococcus, (I) Kurthia, and (J) Pelomonas. (K) Heatmap of cluster stacking at the genus level. The data are expressed as the means ± SDs (n = 6 for all the groups) (*p < 0.05, **p < 0.01, ***p < 0.001, ****p < 0.0001 BLM vs. Control; #p < 0.05, ##p < 0.01, ###p < 0.001, ####p < 0.0001 vs. BLM). [file Image_2.tif]

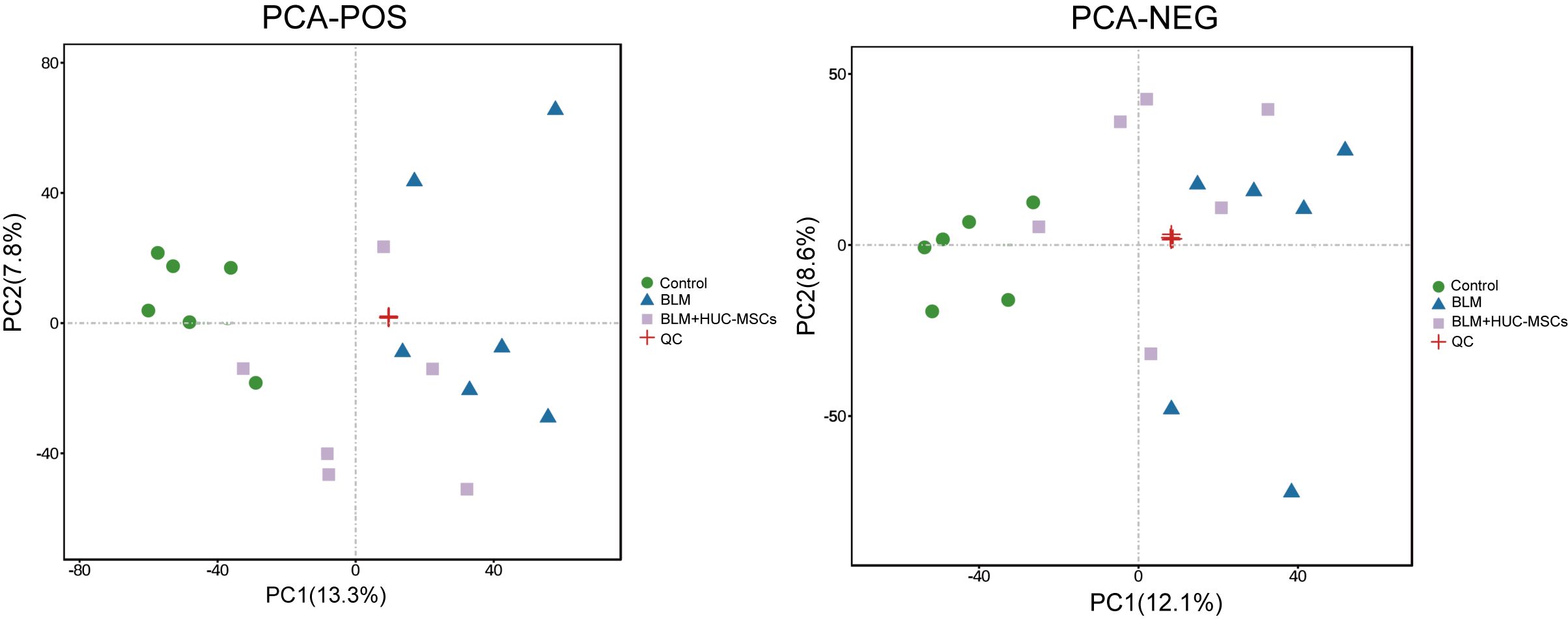

Supplement: Supplementary Figure 3 — PCA score plot of three different groups in positive and negative ion modes (including QC samples). [file Image_3.tif]
